# Perturbation Theory for Scattering from Multilayers with Randomly Rough Fractal Interfaces: Remote Sensing Applications

**DOI:** 10.3390/s18010054

**Published:** 2017-12-27

**Authors:** Pasquale Imperatore, Antonio Iodice, Daniele Riccio

**Affiliations:** 1 Istituto per il Rilevamento Elettromagnetico dell’Ambiente, Consiglio Nazionale delle Ricerche (CNR), 80124 Napoli, Italy; imperatore.p@irea.cnr.it; 2 Dipartimento di Ingegneria Elettrica e delle Tecnologie dell’Informazione, Università di Napoli Federico II, 80125 Napoli, Italy; antonio.iodice@unina.it

**Keywords:** electromagnetic scattering, layered media, fractals, remote sensing

## Abstract

A general, approximate perturbation method, able to provide closed-form expressions of scattering from a layered structure with an arbitrary number of rough interfaces, has been recently developed. Such a method provides a unique tool for the characterization of radar response patterns of natural rough multilayers. In order to show that, here, for the first time in a journal paper, we describe the application of the developed perturbation theory to fractal interfaces; we then employ the perturbative method solution to analyze the scattering from real-world layered structures of practical interest in remote sensing applications. We focus on the dependence of normalized radar cross section on geometrical and physical properties of the considered scenarios, and we choose two classes of natural stratifications: wet paleosoil covered by a low-loss dry sand layer and a sea-ice layer above water with dry snow cover. Results are in accordance with the experimental evidence available in the literature for the low-loss dry sand layer, and they may provide useful indications about the actual ability of remote sensing instruments to perform sub-surface sensing for different sensor and scene parameters.

## 1. Introduction

The problem of scattering of electromagnetic waves from natural stratifications has attracted much interest in recent decades, due to the crucial role that it plays in the remote sensing arena. In this framework, the most general scheme that can be adopted to model the superficial structure of the Earth consists of (piecewise) layered random media [1,2]. Subsurface structures analysis using remote sensing has important repercussions from an applications perspective (hydrology, geology, archaeology, etc.). However, due to the complexity of electromagnetic wave interaction with such natural structures, the scattering behavior of natural media with interfacial roughness remains poorly understood, particularly concerning the implications for radar remote sensing. For such a purpose, a quantitative mathematical analysis of wave propagation in 3-D layered rough media is of crucial importance in order to understand scattering phenomena in such structures. The potential detectability of natural sub-surface layers is intimately related to the availability of such a tool.

This problem has been investigated following different approaches. While some authors have adopted different numerical methods, most of them have addressed two-dimensional geometries [3,4,5,6]. However, the associated computational burden can be prohibitive, hindering their practical application. In addition, a numerical approach can provide specific prediction for a prescribed scenario only, and accordingly it cannot capture the relevant functional dependency on the involved parameters. This point is also crucial in the perspective of retrieving added-value information from the radar data.

Empirical models have been also adopted (see, for instance [1,2]); however, the remote sensing application of this approach remains questionable, as it is neither rigorous nor reliable due to the inherent empiricism involved.

Analytical methods allow us to overcome such limitations, providing approximate expressions for relevant scattering problem. The analytic solutions, which are valid within their domain of applicability, offer a direct functional dependence on the parameters and, in some cases, they give insight in the physics of the problem.

A high-frequency analytical method based on the Kirchhoff-tangent plane approximation (KA) and the geometric optics approximation for the analysis of two rough interfaces has only been proposed in [7,8].

Another approach relies on the perturbation theory [9,10,11,12,13,14,15,16,17,18,19,20,21]. Within this framework, two different systematic formulations have been recently introduced to deal with the analysis of a layered structure with an arbitrary number of rough interfaces. Specifically, the results of the *Boundary Perturbation Theory* (BPT) [12,13,14,15] lead to polarimetric, formally symmetric, and physically revealing closed-form analytical solutions. In this case, a suitable perturbation pertinent to the structure geometry is concerned. The *volumetric-perturbative reciprocal theory* (VPRT) for the evaluation of the scattering from a layered structure with an arbitrary number of rough interfaces considers a perturbation pertinent to the dielectric properties of the structure [18]. It is important to note that both BPT and VPRT lead to formally identical expressions of the first-order scattered field. A complete physical interpretation of the relevant analytical solution has been presented in [16]. A comprehensive discussion on the relevant domain of applicability is also provided in [21]. Note that also higher-order perturbative solutions are available [19,22,23,24], but they require the numerical evaluation of integrals, thus losing most of the advantages of analytical methods mentioned above. Accordingly, we here focus on first-order solutions.

A further consideration is in order. Models derived in the framework of the radiative transfer theory [25,26,27], due to the many simplifications, turn out to be typically not polarimetric. In addition, physical-optics-based models are not rigorously polarimetric, insofar as they employ the non-rigorous tangent-plane approximation. On the contrary, perturbation-theory-based models, being analytically derived from the Maxwell’s equations, come out, accordingly, polarimetric in principle (although at the first order they cannot account for cross-polarization in the backscattering case).

With regard to rough interfaces, it has been broadly recognized that fractal geometry is the most appropriate tool for the description of natural interfaces [28,29,30,31,32]. In particular, interfacial corrugations of natural layered media can be characterized by employing non-stationary processes with stationary increments, since they exhibit multi-scale behavior, self-affinity, increasing variance with scan length, and power spectral functions following a power law. The fractal behavior of natural interfaces is strictly related to the underlying physics of the processes (erosion, fluid invasion of porous media, etc.) that produce such a roughness, but, in spite of that, in electromagnetic scattering problems, interfacial roughness in layered media are usually described by using classical (non-fractal) models. In fact, although the problem of scattering from natural rough surfaces has been deeply investigated in the literature by jointly applying fractal geometry and approximated scattering methods, such as classical Kirchhoff and SPM approximations (for a comprehensive treatment, see [29]), in remote sensing application contexts, the analysis of scattering from a natural layered structure is less advanced, in spite of the widely recognized crucial role of self-affine fractal scaling in description of surfaces and interfaces occurring in nature. To the best of our knowledge, fractal models for descriptions of random rough interfaces addressed to radar signature evaluation of geophysical multilayer structures have not been considered yet by other authors, and we ourselves have only briefly proposed it in a conference paper [33].

In this paper, we investigate the geoscience application of perturbation theory for the characterization of radar response patterns of natural rough multilayers. The aim is to systematically show how the perturbative model can be successfully applied to several situations of interest for microwave remote sensing, since it provides a rationale to understand the basic scattering properties of natural rough multilayers, in particular when lower frequency bands are used, for which interface roughness satisfies validity limits of the perturbative approach. In order to do this, we apply the developed perturbation theory to fractal interfaces and, at variance with what we presented in [33], we here consider actually existing natural structures [34,35,36], representative of two classes of natural stratifications: wet paleosoil covered by a low-loss dry sand layer and a sea-ice layer above water with dry snow cover.

The paper is organized as follows. First, the foundation of the theoretical model is introduced. Numerical examples are then presented and discussed with reference to the layered structure of particular interest for remote sensing, showing a consistent and plausible explanation of the NRCS dependency on the several involved parameters.

## 2. Methods

In this section, we present the BPT (or VPRT) scattering solution for mono-static radar configuration; in addition, a proper characterization of the relevant interfacial roughness is adopted by resorting to the fractional Brownian motion (fBm) fractal process.

### 2.1. Normalized Radar Cross Section (NRCS)

**Figure 1:**
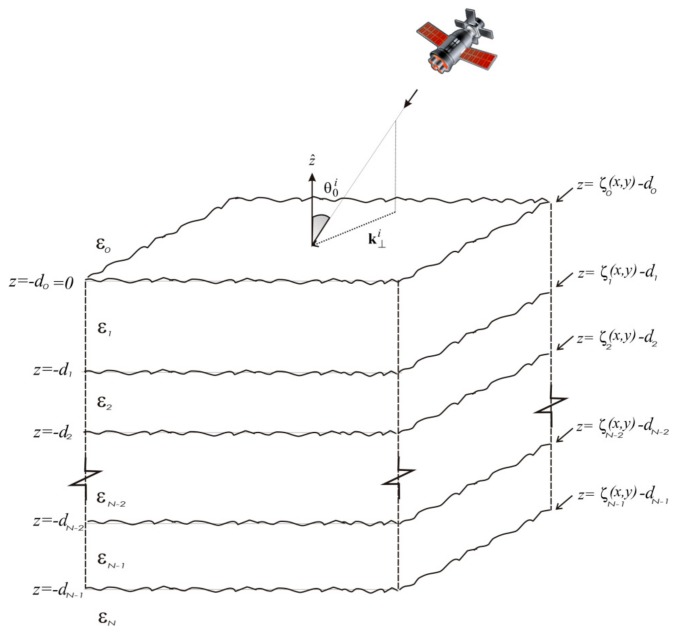
Geometry of the backscattering problem.

The BPT compact closed-form solution was derived in the perturbative first-order limit in [12,13,14,15], for the general bi-static configuration, to model polarimetric wave scattering from 3-D layered structures with an arbitrary number of gently rough interfaces (Figure 1). The same result can be obtained using VPRT [18], but for the sake of conciseness in the following, we will refer to this formulation as a “BPT solution.” Figure 1 illustrates the geometry of the problem: each layer is assumed to be homogeneous and characterized by the dielectric relative permittivity *ε_m_* and the thickness ∆*_m_* = *d_m_* − *d_m_*_−1_. The latter are treated as deterministic parameters, whereas the rough 2-D height profiles of the interfaces are modeled as wide-sense stationary (i.e., statistically homogeneous) 2-D stochastic processes. The general solution of [12,13,14,15] is here specialized to the backscattering configuration, of interest for most remote sensing applications. It is worth noting that, in the backscattering case, BPT cross-polarized scattering coefficients evaluated in the incidence plane vanish, in agreement with the classical first-order SPM method for a rough surface between two different media.

In order to introduce the BPT solution, let us preliminarily define the *generalized reflection coefficients* ${ℜ}_{m-1|m}^{p}$, for the *p*-polarization (*h* or *v*), at the interface between the regions (*m* − 1) and *m*, as the ratio of the amplitudes of upward- and downward-propagating waves immediately above the interface. They can be recursively expressed as [14]

(1) $${ℜ}_{m-1|m}^{p}(k_{⊥}^{i})=\frac{R_{m-1|m}^{p}(k_{⊥}^{i})+{ℜ}_{m|m+1}^{p}(k_{⊥}^{i})e^{j2k_{zm}^{i}\Delta_{m}}}{1+R_{m-1|m}^{p}(k_{⊥}^{i}){ℜ}_{m|m+1}^{p}(k_{⊥}^{i})e^{j2k_{zm}^{i}\Delta_{m}}}.$$

In addition, we define the *generalized transmission coefficients* in the downward direction ${ℑ}_{0|m}^{p}$ as

(2) $${ℑ}_{0|m}^{p}(k_{⊥}^{i})=\;\;\exp\left[j\sum_{n=1}^{m-1}k_{zn}^{i}\Delta_{n}\right]\;\prod_{n=0}^{m-1}\;T_{n|n+1}^{p}(k_{⊥}^{i}){\left[\prod_{n=1}^{m}(1-R_{n|n-1}^{p}(k_{⊥}^{i}){ℜ}_{n|n+1}^{p}(k_{⊥}^{i})e^{j2k_{zn}^{i}\Delta_{n}})\right]}^{-1}.$$

In Equations (1) and (2), $T_{m|m+1}^{p}(k_{⊥}^{i})$ and $R_{m|m+1}^{p}(k_{⊥}^{i})$ represent the *ordinary* transmission and reflection coefficients at the interface between the regions *m* and *m* + 1 evaluated at the incident direction univocally identified by $k_{⊥}^{i}$, the modulus of the vector $k_{⊥}^{i}=k_{x}^{i}\hat{x}+k_{y}^{i}\hat{y}$, i.e., of the two-dimensional projection of incident wave-number vector on the plane *z* = 0; in addition, $k_{zm}^{i}=\sqrt{k_{m}^{2}-{\left|k_{⊥}^{i}\right|}^{2}}=k_{m}\cos\theta_{m}^{i}$, with *k_m_* being the propagation constant in the *m*-th layer.

The global *normalized radar cross section* (NRCS) of the *N*-rough interface layered media can be then expressed as

(3) σ˜pp0=π k04 ∑n=0N−1|α˜ppn,n+1(k⊥i)|2Wn(−2k⊥i)+π k04 ∑i≠j Re { α˜ppi,i+1(k⊥i)(α˜ppj,j+1(k⊥i))*}  Wij(−2k⊥i)

where *p* ∊ {*v*, *h*} indicates the incident polarization, the asterisk symbolizes the complex conjugated, $W_{n}(\kappa )$ is the (spatial) *power spectral density* of the *n*-th corrugated interface, $W_{ij}(\kappa )$ is the *cross power spectral density* between the interfaces *i* and *j*, and

(4) $${\tilde{\alpha }}_{hh}^{m,m+1}(k_{⊥}^{i})=-(\varepsilon_{m+1}-\varepsilon_{m})\;{\left(\xi_{0\to m}^{+h}(k_{⊥}^{i})\right)}^{2}$$

(5) $${\tilde{\alpha }}_{vv}^{m,m+1}(k_{⊥}^{i})=(\varepsilon_{m+1}-\varepsilon_{m})\;\left[\frac{\varepsilon_{m}}{\varepsilon_{m+1}}{\left(\frac{k_{⊥}^{i}}{k_{0}\varepsilon_{m}}\xi_{0\to m}^{+v}(k_{⊥}^{i})\right)}^{2}+{\left(\frac{k_{zm}^{i}}{k_{0}\varepsilon_{m}}\xi_{0\to m}^{-v}(k_{⊥}^{i})\right)}^{2}\right]$$

wherein

(6) $$\xi_{0\to m}^{\pm p}(k_{⊥}^{i})={ℑ}_{0|m}^{p}(k_{⊥}^{i})\;e^{jk_{zm}^{i}\Delta_{m}}[1\pm {ℜ}_{m|m+1}^{p}(k_{⊥}^{i})].$$

Accordingly, the presented closed-form solution allows for the evaluation of backscattering from the layered rough structure, as a function of the parameters of the three-dimensional layered structure (layers thickness, complex permittivities, and shape of the roughness spectra) and those of the incident field (frequency, polarization, and direction of incidence). Thus, the BPT solution parametrically accounts for the dependence of scattering properties on the layered structure geometric and electromagnetic parameters. In addition, the NRCS of the layered media is sensitive to the correlation between rough profiles of different interfaces. In particular, when completely uncorrelated random interfaces are concerned, scattered intensity (in the first-order approximation) arises from the incoherent superposition of the field contributions scattered from each rough interface. Finally, it can be noted that all previous existing perturbative scattering models, introduced by other authors to deal with some simplified layered geometry with one or two rough interfaces, can be all rigorously regarded as special cases of the general BPT solution [12,13,14].

### 2.2. Interface Roughness Description: 2-D fBm

A stochastic process $\zeta_{m}=\zeta_{m}(r_{⊥})=\zeta_{m}(x,y)$ is an fBm surface if its increments $\zeta_{m}(x,y)-\zeta_{m}(x^{\prime },y^{\prime })$ over any distance $\tau =\sqrt{{\left(x-x^{\prime }\right)}^{2}+{\left(y-y^{\prime }\right)}^{2}}$ are Gaussian with variance $s^{2}\tau^{2H}$ [28,29], where *H* is the *Hurst* coefficient and *s* is a real parameter measured in [m^(1−*H*)^]. It can be demonstrated that, for 0 < *H* < 1, such a process exists, that it is statistically self-affine, and that its sample surfaces have fractal dimension *D* = 3 − *H* with a probability of 1 [28]. In addition, the parameter *s* is linked to a characteristic length of the fBm surface, called topothesy *T*, by the relation $s=T^{\left(1-H\right)}$. *Topothesy* is defined as the distance over which chords joining points on the surface have a root mean square (rms) slope equal to unity. Furthermore, the power spectrum of an fBm two-dimensional process is

(7) $$W_{m}(\kappa )=S_{o}\kappa^{-\alpha }$$

where α=2+2H=8−2D, $\kappa =\sqrt{\kappa_{x}^{2}+\kappa_{y}^{2}}$ is the spatial frequency ($\kappa_{x}^{}$ and $\kappa_{y}^{}$ being the Fourier mates of *x* and *y*, respectively), and *S*_0_ is the spectral parameter (*S*_0_ > 0), measured in [m^(2−2*H*)^]. Additionally, from the inequalities 0 < *H* < 1, we get 2 < *α* < 4. The relation between the spatial parameter *s* and the spectral one *S*_0_ is given by

(8) $$s^{2}=\frac{S_{0}}{2\pi H}2^{-2H}\frac{\Gamma \left(1-H\right)}{\Gamma \left(1+H\right)}.$$

Γ(x) being the Gamma function. A 2-D stochastic process $\zeta_{m}(r_{⊥})$ satisfying above fBm definition for any value of *τ* is sometimes referred to as *mathematical* fBm. It is only locally statistically homogeneous, and it shows infinite variance. However, in scattering problems, it is sufficient that $\zeta_{m}(r_{⊥})$ satisfies the above definition on a range of scales *τ*_min_ < *τ* < *τ*_max_, where *τ*_max_ is of the order of the linear size of the illuminated volume (i.e., of the order of the radar sensor resolution) and *τ*_min_ is of the order of a fraction of the incident wavelength *λ*, say *λ*/10. Such a process is sometimes called a *physical* fBm. It has finite variance and is band-limited and statistically homogeneous [29]. Therefore, the BPT solution of Section 2.1 can be also applied to a rough multilayer whose interfacial roughness is described by using a physical fBm process.

### 2.3. Validity Limits

As already mentioned, mathematical fBm surfaces are self-affine at all scales, so that they have details on any arbitrary small scale [29]. Conversely, physical fBm exhibits a fractal behavior limited to a range of scales. For such band-limited fBm processes, relations between fractal and classical surface parameters can be established [29]; they are summarized in Table 1. We highlight that it turns out that the height standard deviation is proportional to the length of the considered profile raised to a power smaller that unity. This is a key-point when natural interface modeling is concerned.

The domain of validity of the BPT solution in terms of the classical parameters characterizing each interface is discussed in depth in [21], and can be summarized as follows. The height standard deviation of the rough interfaces, about the unperturbed interface, must be small compared to the wavelength in the medium, and the standard deviation of the gradient of the interface must be small in comparison to unity. As a result, the classical validity conditions can be transferred in terms of fractal parameters of the interfaces, according to the relations in Table 1.

## 3. Results and Discussion

In this section, we present some numerical examples aimed at analyzing the scattering behavior of geophysical layered structures with rough interfaces. To this aim, we consider a layered medium with three rough interfaces, which is representative for the near surface stratigraphy of several situations of interest, and whose parametric characterizations will be provided in the following, based on real natural stratifications described in past literature. A parametric analysis at the L-band (1.6 GHz) and C-band (5.3 GHz) of the polarimetric signatures, based on the perturbative scattering model predictions, is provided for typical geophysical structural parameters characterizing different natural scenarios. We also explore the sensitivity of radar signature to the model parameters.

### 3.1. Sub-Surface Sensing of Wet Paleosoil Covered by a Low Loss Dry Sand Layer

This first case study is addressed to investigate the capability of L-band radar to penetrate soils for retrieving information about subsurface wet structures. Specifically, we refer to bare sandy layer covering wet subsurface structures (paleosoils) [34]. Accordingly, we consider a layered structure with only two rough interfaces. At 1.6 GHz, we assume *ε*_1_ = 3.4 + *j*0.02 (sand permittivity) and *ε*_2_ = 14.2 + *j*1.064 (permittivity of Paleosoil). With respect to a dry material such as sand, buried paleosoil presents a higher permittivity due to its water content. The description of the natural roughness of each interfacial is obtained by adopting an fBm process. As discussed in Section 2, the fractal model prescribes a power-law behavior at any scale (see (8)), and the two fractal parameters allow us to select the shapes of these power-law behaviors. The roughness of the upper interface is characterized by the fractal parameters *H* = 0.78 and *S*_0_ = 1.6 × 10^−4^ [m^(2−2*H*)^], and the lower interface is characterized by *H* = 0.75 and *S*_0_ = 2.1 × 10^−4^ [m^(2−2*H*)^]. In this way, the air–sand interface is assumed smoother than the sand–paleosoil one. The Hurst coefficient *H* is related to the fractal dimension of the surface. It should be noted that the lower the *H* value, the higher the fractal dimension. Moreover, we suppose no correlation between the interfaces. It can be verified that, with these fractal parameters and wavelength, and for sensor resolutions (*τ*_max_) of a few meters, validity limits of BPT-fBm model, see Section 2.3, Table 1, are satisfied. In particular, for a resolution of 3 m, the roughness height standard deviation is about 8 mm (while wavelengths in the media are about 20 cm, 13 cm, and 8 cm). In addition, the standard deviation of the gradient of the roughness, for *τ*_min_ = *λ*/10, is about 0.005.

**Figure 2:**
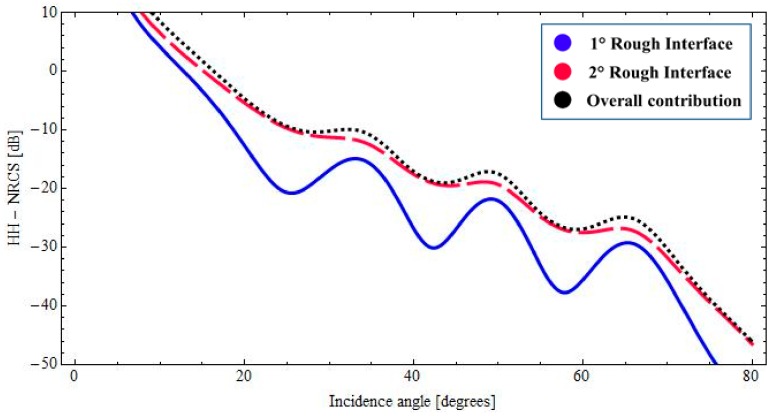
*BTP*-fBm model of a layered medium with two fractal interfaces. Backscattered HH NRCS as a function of incidence angle at 1.6 GHz: *ε*_1_ = 3.4 + *j*0.020 (sand permittivity), *ε*_2_ = 14.2 + *j*1.064 (paleosoil permittivity), sand thickness ∆_1_ = 1.20 [m].

**Figure 3:**
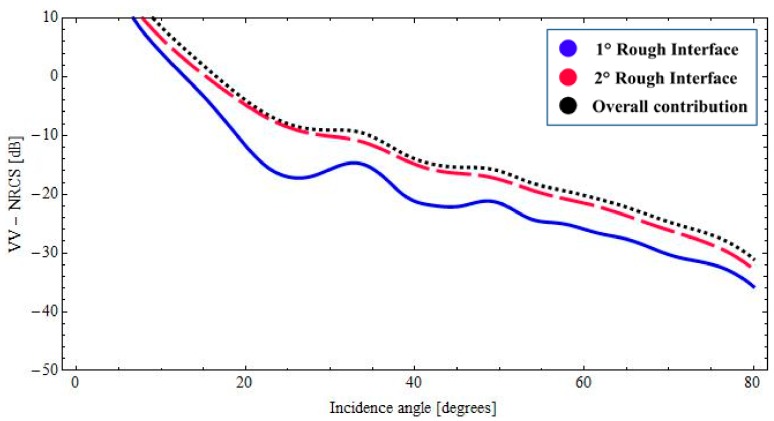
*BTP*-fBm model of a layered medium with two fractal interfaces. Backscattered VV NRCS as a function of incidence angle at 1.6 GHz: *ε*_1_ = 3.4 + *j*0.020 (sand permittivity), *ε*_2_ = 14.2 + *j*1.064 (paleosoil permittivity), sand thickness ∆_1_ = 1.20 [m].

With reference to the geophysical structure described above, in Figure 2 and Figure 3, we show the backscattered NRCS, as predicted by the BPT-fBm model, as a function of incidence angle for the horizontal (HH) and vertical (VV) polarization, respectively. We have assumed a sand thickness ∆_1_ = 1.20 [m] in both cases. In Figure 2 and Figure 3, the contributions of the first and second fractal interfaces are represented, respectively, by the solid (blue) line, the long-dashed (red) line. In addition, the overall contribution is also depicted (dotted black line). As we can recognize from the graphs of Figure 2 and Figure 3, total backscattering is mainly generated by the sand–paleosoil interface. These results are in accordance with the experimental evidence obtained in [1,2,34]. A more quantitative comparison with these experimental results would be desirable. However, in [1], no roughness information is provided. In [2,34], as in most electromagnetic scattering experiments, the surface roughness is described in terms of classical parameters (standard deviation and correlation length), which cannot be univocally translated in fractal parameters *S*_0_ and *H* without knowledge of the size of the illuminated area (see [29]). Accordingly, no fully quantitative comparison can be performed with these results, and only the qualitative one reported here is possible.

### 3.2. Wet Paleosoil Covered by a Low Loss Dry Sand Layer: Sensitivity of Radar Signature to the Model Parameters 

**Figure 4:**
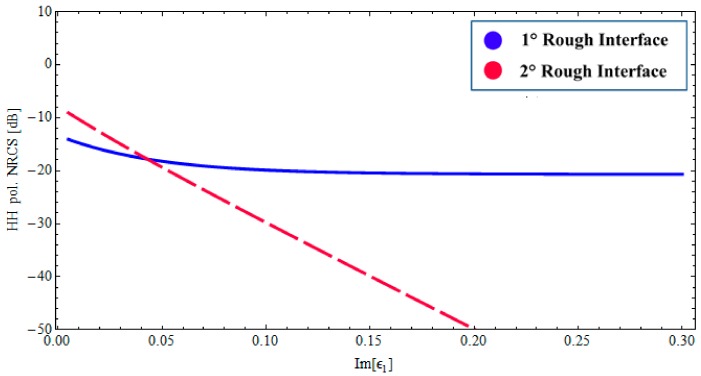
*BTP*-fBm model of a layered medium with two fractal interfaces. Backscattered HH NRCS as a function of as a function of imaginary part of the complex permittivity *ε*_1_ at 1.6 GHz: incidence angle $\theta_{0}^{i}=$ 35°, Re[*ε*_1_] = 3.4 (sand permittivity), *ε*_2_ = 14.2 + *j*1.064 (paleosoil permittivity), sand thickness ∆_1_ = 1.20 [m].

**Figure 5:**
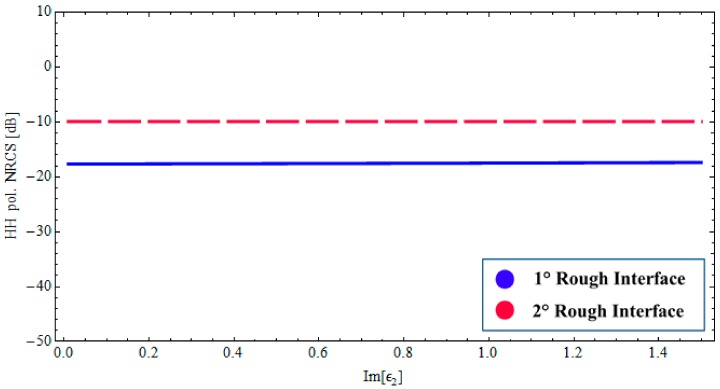
*BTP*-fBm model of a layered medium with two fractal interfaces. Backscattered HH NRCS as a function of as a function of imaginary part of the complex permittivity *ε*_2_ at 1.6 GHz: incidence angle $\theta_{0}^{i}=$ 35°, *ε*_1_ = 3.4 + *j*0.020 (sand permittivity), Re[*ε*_2_] = 14.2 (paleosoil permittivity), sand thickness ∆_1_ = 1.20 [m].

We first explore the dependency on the losses in the sand layer, which is crucial for subsurface structure sensing. Accordingly, we analyze the backscattered NRCS, as predicted by the BPT-fBm model, as a function of imaginary part of the complex permittivity *ε*_1_. This dependency is depicted in Figure 4, for a fixed incidence angle ($\theta_{0}^{i}=$ 35°) at 1.6 GHz, and the horizontal (HH) polarization case only. We assume the same fractal description as in Figure 2 and Figure 3. In Figure 4, the contributions of the first and second fractal interfaces are represented, respectively, by the solid (blue) line and the long-dashed (red) line. As can be seen, the sensitivity to the losses in the sand layer is significantly different for the scattering contributions pertinent to the air–sand and sand–paleosoil fractal interfaces. In particular, as the losses in the sand layer increase, the scattering contribution associated with the buried interface decreases rapidly, whereas the upper-interface contribution remains essentially unaffected. This behavior is closely related to the inherent penetration depth. Conversely, the radar signature is not affected by losses variation (within the physically meaningful range) in the wet paleosoil, as is evident in Figure 5.

**Figure 6:**
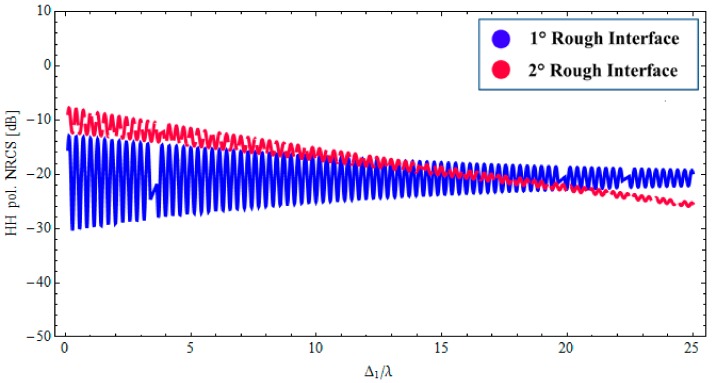
*BTP*-fBm model of a layered medium with two fractal interfaces. Backscattered HH NRCS as a function of as a relative thickness of the sand layer at 1.6 GHz: incidence angle $\theta_{0}^{i}=$ 35°, *ε*_1_ = 3.4 + *j*0.020 (sand permittivity), *ε*_2_ = 14.2 + *j*1.064 (paleosoil permittivity).

The radar response also exhibits a certain dependence on the real part of the permittivities of sand and wet paleosoil, which is not shown here to save space. How the thickness of the sand layer affects the contribution of the two rough interfaces is shown in Figure 6. It should be noted that, in this case, the lower interface is dominant up to few meters (2–3 m). These results confirm that the L-band SAR can explore the buried wet paleosoil structure down to several meters when covered by dry material such as sand. We stress also that the different degree of roughness of the two interfaces obviously influences the radar signature of the structure.

Finally, it is worth noting that the low losses in the top dry sand layer allow for the presence of coherent interference among multi-bounce contributions within the layer: this is evidenced by the oscillations in plots of Figure 2, Figure 3 and Figure 6.

### 3.3. Remote Sensing of a Sea-Ice Layer above Water with Dry Snow Cover

Analysis of backscattering form Arctic and Antarctic sea ice can be properly modeled by adopting a rough multilayer structure. The structure here considered is composed of an upper layer representing a dry snow cover, a middle layer (sea ice), and the lower half space is homogeneous water [35,36]. Specifically, the considered vertical profile is characterized by the following parameters: *ε*_0_ = 1.0, *ε*_1_ = 1.65 + *j*0.064, *ε*_2_ = 3.38 + *j*0.15, *ε*_3_ = 60.43 + *j*40.5; Δ_1_ = 0.07 [m], Δ_2_ = 0.2 [m].

**Figure 7:**
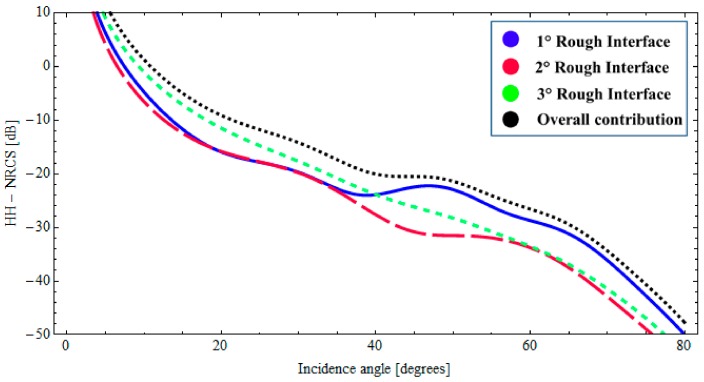
*BTP*-fBm model of a layered medium with three fractal interfaces. Backscattered HH NRCS as a function of incidence angle at 5.3 GHz: *ε*_0_ = 1.0 (air), *ε*_1_ = 1.65 + *j*0.064 (snow cover), *ε*_2_ = 3.38 + *j*0.15 (sea ice), *ε*_3_ = 60.43 + *j*40.5 (water); Δ_1_ = 0.07 [m], Δ_2_ = 0.2 [m].

**Figure 8:**
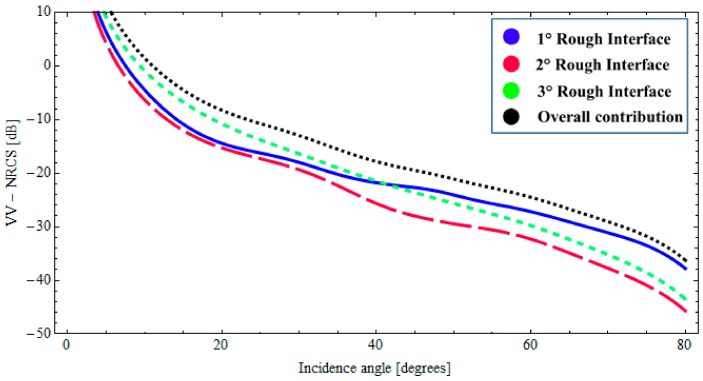
*BTP*-fBm model of a layered medium with three fractal interfaces. Backscattered VV NRCS as a function of incidence angle at 5.3 GHz: *ε*_0_ = 1.0 (air), *ε*_1_ = 1.65 + *j*0.064 (snow cover), *ε*_2_ = 3.38 + *j*0.15 (sea ice), *ε*_3_ = 60.43 + *j*40.5 (water); Δ_1_ = 0.07 [m], Δ_2_ = 0.2 [m].

**Figure 9:**
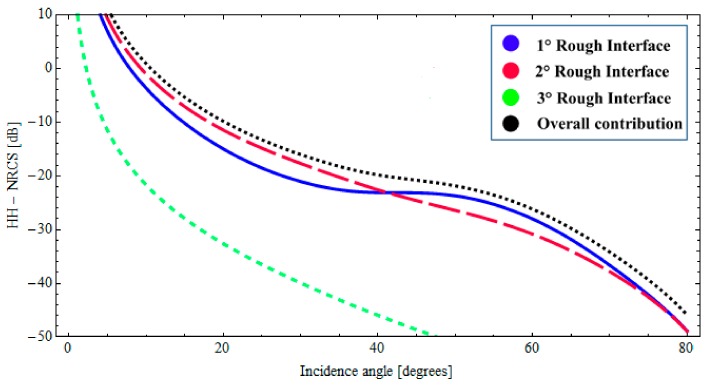
*BTP*-fBm model of a layered medium with three fractal interfaces. Backscattered HH NRCS as a function of incidence angle at 5.3 GHz: *ε*_0_ = 1.0 (air), *ε*_1_ = 1.65 + *j*0.064 (snow cover), *ε*_2_ = 3.38 + *j*0.25 (sea ice), *ε*_3_ = 60.43 + *j*40.5 (water); Δ_1_ = 0.07 [m], Δ_2_ = 0.2 [m].

**Figure 10:**
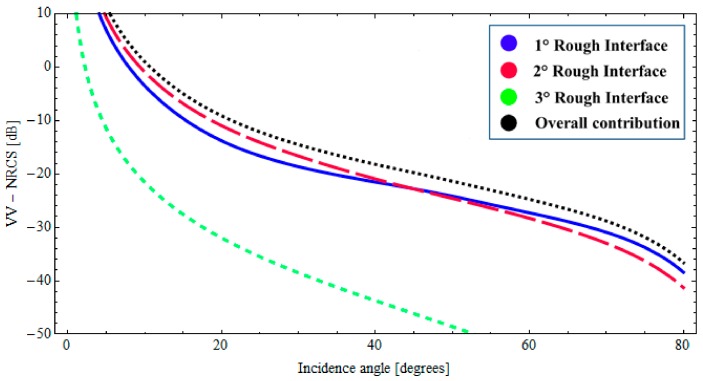
*BTP*-fBm model of a layered medium with three fractal interfaces. Backscattered VV NRCS as a function of incidence angle at 5.3 GHz: *ε*_0_ = 1.0 (air), *ε*_1_ = 1.65 + *j*0.064 (snow cover), *ε*_2_ = 3.38 + *j*0.25 (sea ice), *ε*_3_ = 60.43 + *j*40.5 (water); Δ_1_ = 0.07 [m], Δ_2_ = 0.2 [m].

In this case, the description of the natural roughness of each interface is also obtained by adopting an fBm process. The roughness of the upper interface is characterized by the fractal parameters *H* = 0.70 and *S*_0_ = 1.9 × 10^−4^ [m^(2−2*H*)^], the intermediate interface is characterized by *H* = 0.78 and *S*_0_ = 1.6 × 10^−4^ [m^(2−2*H*)^], the fractal parameters of the lower interface are *H* = 0.75 and *S*_0_ = 2.1 × 10^−4^ [m^(2−2*H*)^]. Moreover, we suppose no correlation between the interfaces. The evaluation of the backscattered NRCS is then obtained by applying the BPT-fBm model to this canonical structure. It can be verified that, with these fractal and dielectric parameters, at 5.3 GHz and for sensor resolutions of a few meters, the validity limits of the BPT-fBm model (see Section 2.3) are certainly satisfied with regard to the roughness gradient, which is smaller than 0.01. The roughness height standard deviation is still less than 1 cm, so the condition on roughness standard deviation is satisfied for the first layers, but, due to the high dielectric constant and hence short wavelength, not for the water layer. However, we believe that the obtained results are still a reasonable estimate of backscattering, especially when (as in the second of the cases discussed in the following) the scattering contribution from the ice–water interface is negligible compared to that from other interfaces. In Figure 7 and Figure 8, the backscattered NRCS of the three-interfaces stratification at 5.3 GHz is shown as a function of incidence angle, for both horizontal (HH) and vertical (VV) polarizations, respectively. In Figure 7 and Figure 8, the contributions of the first, second, and third rough interfaces are represented, respectively, by a solid (blue) line, a long-dashed (red) line, and a short-dashed (green) line. In addition, the overall contribution is also depicted (dotted black line). It can be noted from Figure 7 and Figure 8 that, due to the large dielectric discontinuity at the ice–water boundary, in this case, the dominant contribution arises from a lower interface insofar as the incident angle is less than 40°. Nonetheless, when the losses in the ice layer increases, the radar signature can become dominated by scattering from the snow–ice interface. This is demonstrated in Figure 9 and Figure 10, in which we assume a different permittivity of the sea-ice layer, ε_2_ = 3.38 + *j*0.25, and a greater degree of roughness of the snow–ice interface, *H* = 0.70 and *S*_0_ = 1.9 × 10^−4^ [m^(2−2*H*)^]. In this case, the contribution of the lower rough interface is negligible due to the relevant absorption in the ice, whereas the backscattering contributions pertaining to the intermediate interface (snow–ice) is dominant, insofar as the incident angle is less than 40°.

It is worth noting that the relevance of the scattering contributions due to the different interfaces can also vary according to their respective amount of roughness. This dependency is naturally taken into account by the BPT-fBm model. It is also important to note that, although the relative degree of roughness of the interfaces can significantly affect the final radar signature, a precise characterization of the relevant roughness can be in general unavailable. It should be also noted that, even if the contribution of the lower rough interface is not essential, the global return nevertheless can be affected significantly by the characteristics of the underlying strata.

As a result, these simple examples demonstrate that neglecting subsurface structure can be unrealistic. Therefore, the BPT-fBm model provides a powerful tool for understanding the dependency of the backscattering of natural stratification on the different parameters involved.

## 4. Conclusions

We have analyzed the electromagnetic backscattering from natural rough layered structures. In particular, we have proposed the joint use of the *BPT* closed-form scattering solution and the fBm fractal interfacial description and have shown that it provides a unique tool for the characterization of radar response of natural rough multilayers. In addition, we have presented and discussed numerical examples relative to some real-world layered structures of practical interest in remote sensing applications. It turns out that the BPT-fBm model provides a consistent explanation of the NRCS dependency on the several involved parameters, thus providing a rationale for the comprehension of the basic scattering properties of natural rough layered structures.

We finally underline that future work is certainly desired to extend the validity of the approach to account also for cross-polarized backscattering. This could be obtained by resorting to higher-order perturbative solutions; however, as underlined in the introduction, this would require the numerical evaluation of integrals, thus losing most of the advantages of analytical methods. A more convenient way would be to resort to the two-scale polarimetric approach; the case of a single interface was investigated in [37]. However, an extension of that approach to the case of fractal multilayers is beyond the scope of this manuscript and can be a subject of future work. Similarly, inclusion of volumetric scattering would be desirable, for instance, to model the effect of snow grains in snow layers; however, this is by no means straightforward and is another potential subject for future work.

## Figures and Tables

**Table 1:** Variance of interface height and slope for band-limited fBm processes.

Interface Height Variance	$\sigma^{2}≅\frac{S_{0}}{4\pi \;H}\tau_{\max}^{2H}$
Interface Slope Variance	$\sigma \prime {}^{2}≅\frac{S_{0}}{4\pi (1-H)}\tau_{\min}^{2H-2}$
